# Matrices of Different Natures for Bone Tissue Engineering—A Comparative Analysis

**DOI:** 10.3390/ma18184244

**Published:** 2025-09-10

**Authors:** D. Ya. Aleinik, A. E. Bokov, D. D. Linkova, E. A. Levicheva, E. A. Farafontova, R. S. Kovylin, V. V. Yudin, D. V. Khramova, L. A. Cherdantseva, S. A. Chesnokov, I. A. Kirilova, M. N. Egorikhina

**Affiliations:** 1 Federal State Budgetary Educational Institution of Higher Education, Privolzhsky Research Medical University of the Ministry of Health of the Russian Federation, Nizhny Novgorod 603005, Russia; daleynik@yandex.ru (D.Y.A.); andrei_bokov@mail.ru (A.E.B.); linckovadaria@yandex.ru (D.D.L.); kate.lekat@yandex.ru (E.A.L.); ekaterina_farafontova@mail.ru (E.A.F.); yudin@iomc.ras.ru (V.V.Y.); sch@iomc.ras.ru (S.A.C.); 2 G. A. Razuvaev Institute of Organometallic Chemistry of the Russian Academy of Sciences, Nizhny Novgorod 603137, Russia; roman@iomc.ras.ru (R.S.K.); khramovadaria@iomc.ras.ru (D.V.K.); 3 Novosibirsk Research Institute of Traumatology and Orthopedics Named After Ya. L. Tsivyan, Novosibirsk 630091, Russia; lcherdanceva@niito.ru (L.A.C.); irinakirilova71@mail.ru (I.A.K.)

**Keywords:** bone defects, tissue regeneration, bone tissue substitutes, framework, cytotoxicity, cytocompatibility, structure, porosity, polymers

## Abstract

Recent decades have been characterized by increasing numbers of bone tissue injuries and diseases resulting in the formation of bone defects. The number of such bone defects has also grown due to active surgical approaches implemented after surgical interventions for oncological, infectious, and dystrophic bone lesions. To repair such bone defects requires the use of bone tissue substitutes. Nowadays, constructs based on matrices of various compositions and structures, supplemented with the addition of biologically active components (including growth factors and cells), are the most promising approaches used in bone tissue engineering. The properties of the matrices are of the utmost importance in construct formation. This work presents the results of a comprehensive study of matrices of various natures intended for the formation of complex constructs for bone tissue engineering. Using a set of methods for studying the physical, mechanical, and biological characteristics, the total and associated porosity of the studied matrices, the structure, the mechanical parameters, and the level of cytotoxicity and cytocompatibility were determined. It was shown that all the studied materials were not cytotoxic (cytotoxicity rank of all matrices = 0–1). All matrices were porous, but samples of materials of biological origin had large pores ranging in size from 100 to 1000 μm, and pores of the hybrid polymer were sized from 0.1 to 100 μm. Total and open porosity ranged from 89% and 79% for the allogeneic matrix up to 67% and 48% for the hybrid polymer, respectively, while the σ values (compressive stress at break) of samples of all studied materials were close to each other. When human test culture MSCs interact with samples of these materials, it was shown that the cells adhere to the surface and structure of all materials and retain typical morphology, while also demonstrating the ability to proliferate and migrate along the surface and into the matrix structure, i.e., all materials are cytocompatible. Based on the data obtained, it can be assumed that all the studied matrices can be used for model biomedical studies and as a basis for constructs for bone tissue engineering. An adequate choice of research method at the earliest stages of the development of each material will ensure the most effective approaches for further work and subsequent use of this product.

## 1. Introduction

The growing demands of modern medicine require innovative materials to replace bone defects in various parts of the musculoskeletal system. Such defects can be formed both as a result of injuries and wounds, and after surgical treatment of pathological processes in bone tissue of various etiopathogeneses [1,2]. The likelihood of fractures depends greatly on the patient’s age and concomitant pathologies, the majority of which are osteoporosis, endocrine diseases, disorders of mineral metabolism, infections, and bone tumors [3,4,5,6].

There is currently a shortage of suitable bone-substitute materials, due not only to the annual increase in the number of bone injuries and diseases and their severity, but also to the introduction of new techniques and technologies of reconstructive plastic surgery into surgical practice of restorative medicine. Taking into account the particular features of the human skeletal system, consisting of 206 bones with various structures and functions, and the multiple clinical situations that affect the properties of bone tissue, many different types of bone-substitute materials are needed to cover the range of bone tissue restoration requirements. There are plenty of types of such materials, but, to date, none of them can completely copy the structural and functional properties of bone tissue and meet all the requirements of modern clinical practice. Thus, research and the development of new materials and compounds to restore bone structural and functional integrity is ongoing. The demand for bone substitutes is continuously increasing: for instance, in 2023, the global market for bone substitutes was estimated at USD 3.81 billion, and according to forecasts, by 2032, it will have grown to USD 6.67 billion, demonstrating an average annual growth rate of 6.5% during the forecast period from 2024 to 2032 [7].

The materials that are traditionally used to replace bone tissue defects include bone substitutes based either on natural tissues (autologous, allogeneic and xenogenic) or on artificial materials [8,9,10,11]. Autologous grafts remain the “gold standard” for bone tissue defect replacement [12,13]. However, the volume and plasticity of autologous bone tissues are drastically limited, while a further disadvantage of autologous bone grafts is the need for additional surgery, which often results in complications at the site used for sourcing the bone—such as pain, swelling, infection and the risk of damage to adjacent structures [14,15]. Moreover, it is obvious that autologous material cannot be used in all clinical situations.

Allogeneic and xenogenic materials are in high demand for clinical purposes, with the composition and structure of the former being closest to the patient’s own tissues. Moreover, the use of allografts avoids the donor site problems associated with autologous grafts. However, with allografts, there is a greater risk of disease transfer, the rate of engraftment is slower, and the risk of rejection is increased, especially in areas where the blood supply is relatively poor [16]. Further difficulties associated with the use of allografts are the ethical and legal issues related to obtaining donor material, limiting their use as resources.

The most popular xenomaterials are decellularized bone xenografts; their structure being similar to allogeneic grafts, they can create an optimal microenvironment for the infiltration, adhesion, and proliferation of cells both in vitro and for de novo bone tissue formation in vivo. This osteoinductive effect is associated with the preservation of the micro- and macrostructure of the decellularized bone, as well as with the partial retention of the extracellular matrix components [17]. However, despite their advantages of high availability, low cost, and good mechanical and osteoinductive properties, not all xenobone-based materials show positive results in clinical practice [18]. Intensive development of materials science and tissue engineering has recently resulted in the development of composite materials that combine a xenogenic mineral matrix and synthetic and/or natural polymers. Such composite materials have been successfully used clinically [19,20]. Overall, the literature data including long-term experience of the research and application of natural materials enable us to understand both the advantages and disadvantages of each type. In general, it should be noted that natural materials have an inconstant composition and high variability of characteristics, which are determined by the original material or even of its specific batch [21,22,23].

Recently, there has been an increase in the number of research attempts related to the study and application of various polymers as artificial bone tissue substitutes [24,25,26]. This interest in polymeric materials is due to the availability of components, the possibility of obtaining potentially unlimited volumes of them, the availability of basic raw materials, their relatively low cost, ease of storage, the ability to adjust strength characteristics, porosity, the ease and rate of biodegradation, etc. [27,28,29,30].

According to statistics, the synthetic materials segment saw the largest share of the global bone tissue substitutes market in 2023 and is expected to grow at the highest average annual rate during the forecast period (2024 to 2032). The reason for this major share of the segment is attributed to the increasing prevalence of bone-related diseases as well as to the high demand for synthetic products, especially in developed countries. Moreover, the growing number of new products entering the market is expected to contribute to such segment growth during the forecast period [7].

Unfortunately, the use of some of these substitutes does not always ensure complete restoration of the structural and functional properties of bone tissue; thus, complex constructs are being actively developed based both on well-known, well-proven bone substitutes and on new materials with the inclusion of growth factors or various types of cells. Here, the development of tissue-engineered constructs with the use of bone-substituting materials of various origins as a matrix for the cells and with the addition of growth factors is the most promising direction. This trend in regenerative medicine—bone tissue engineering—is developing rapidly and effectively.

The choice of a matrix providing “niches” for stromal cells while also offering appropriate conditions for the migration, proliferation, and differentiation of cells from surrounding tissues is of the utmost importance for successful generation of constructs for use in bone tissue replacement.

Taking into account the huge variety of materials of different composition and structure proposed as matrices for bone tissue substitutes, the study of such materials requires the use of various techniques, in particular, materials science techniques [31]. From the materials science point of view, starting from the initial stages of working with matrix materials, there is a need to provide comprehensive research, including assessment of the physical, mechanical, and biological characteristics that are closely interconnected.

First of all, one should emphasize the importance of such fundamental matrix characteristics as structure and porosity. Porous materials are the most suitable “niches” for development of the necessary microenvironment for stromal cells, providing conditions for the implementation of cellular processes for bone regeneration [32].

Porosity, especially the availability of an interconnected pore system, enhances the adhesive properties of the material and is a required characteristic of the matrix for tissue engineering. It is generally believed that materials for bone implants should have an open porosity of 50–70% with a pore size of at least 100 μm for cell migration into the material and for vascular invasion. An interconnected pore system therefore ensures optimal conditions for colonization of the implanted matrix by osteogenic cells, neovascularization from the surrounding tissues, and the maintenance of cell proliferation and differentiation, forming direct links to the recipient’s bone tissues and gradual replacement with newly formed tissues [33,34]. Hence, right from the start, any assessment of the quality of a potential matrix material for use in forming constructs (bone tissue substitutes) should include a study of the structure and porosity parameters.

It is essential that any new material intended for clinical use must undergo a series of preclinical tests of its biological characteristics before permission for its clinical application is granted. Preclinical studies of biomedical materials are conducted on the basis of international ISO standards and usually begin with in vitro testing. However, it should be noted that while techniques regulated by the DIN, EN, and ISO 10993-5:2009 standards mainly focus on determining the level of cytotoxicity in vitro, the study of bone tissue replacement materials to be used in long-term contact with blood and tissues requires a wider range of studies, including cytotoxicity assessments to ensure there is little interference with cellular processes in vivo.

Analysis of cell behavior on the surface or within the structure of matrix material samples of an in vitro model enables more adequate assessment of the their properties and prediction of the behavior of the construct in clinical conditions. For instance, it becomes feasible to characterize any changes in cell viability occurring directly on the matrix, the capacity of the matrix to provide for adhesion, localization, and appropriate morphology of cells in the material, and changes in their functional activity, by using modern research techniques [35,36,37,38].

Assessment of the above parameters is usually conducted with characterized mammalian cell cultures, in particular human cells. Since specific sensitivity is required when studying bone substitutes intended for long-term contact with blood and tissues, it is preferable to use cell cultures and cell lines obtained for these purposes directly from living tissues [39]. Intelligent and comprehensive use of in vitro models not only allows a reduction in the extent of testing on experimental animals and decreases the overall cost of preclinical studies, but can also predict the likely success of the materials in use.

The aim of the research was to conduct a comparative study of the major structural and functional properties of three matrices for use in bone tissue engineering (two natural matrices and a polymer matrix) and their biological characteristics.

## 2. Materials and Methods

### 2.1. Obtaining Allogeneic Material

The donors of the allogeneic biomaterials provided their informed voluntary consent for its use for research purposes. All donors of the allogeneic biomaterials had no history of tuberculosis, cancer, transmissible infections, or skin diseases.

Cells used as a source of test cultures for research were isolated from biopsies of human skin or adipose tissue obtained in the operating room during planned cosmetic surgery procedures. Adipose tissue biomaterial for cell isolation was only used if the results of screening for blood-borne infections (RW, hepatitis B and C, HIV infection) were negative. The test cultures of human dermal fibroblasts and human adipose tissue mesenchymal stromal cells were obtained and characterized in the biotechnology laboratory of the University Clinic of the aforementioned institute, using methods previously described [40,41].

The bone tissue samples for the allogeneic matrix were obtained from femoral heads resected in patients during surgical procedures scheduled for medical reasons. The tissue material for prepared bone tissue fragments was only used if the results of screening for blood-borne infections (RW, hepatitis B and C, HIV infection) were negative. The prepared bone tissue fragments were subjected to multi-stage sequential decellularization and purification, removing organic components and traces of albumin [1,42]. Sterilization of the finished bone tissue samples was conducted using ionizing radiation on the ILU-10 linear electron accelerator (certificate No. 4/411-0314-18) with a radiation dose of 25 kHz, followed by bacteriological confirmation of sterilization [43].

### 2.2. Determination of Physical and Mechanical Properties

Mercury porosimetry (PASCAL EVO 140/440 ULTRA MACRO porosimeter, Thermo Fisher Scientific, Waltham, MA, USA) was used to determine the pore characteristics of the samples. The structure of sections of the materials was determined by scanning electron microscopy (SEM) using a Regulus SU8100 microscope (Hitachi, Tokyo, Japan) at an accelerating voltage.

The mechanical properties of 10 × 10 × 4 mm blocks of the matrices—cut with a blade—were determined using an Autograph AGX-V 50 universal testing machine with a 5 kN force-measuring cell (Shimadzu, Kyoto, Japan). The strength characteristics (Young’s modulus in compression, E, and the ultimate stress in compression, σ) were determined according to ISO 604:2002 [44].

### 2.3. Determination of Biological Characteristics of the Samples

The levels of cytotoxicity and cytocompatibility of the matrix samples were tested using their interaction with surface-dependent human cells. We studied test cultures of the cells on the matrix samples, looking for changes in their viability, adhesion to the surfaces, proliferation, and migration into the structure of the materials.

### 2.4. MTT Assay

The level (rank) of cytotoxicity was assessed using the MTT assay method [45,46]. For this purpose, the samples were placed in 15 mL centrifuge tubes filled with DMEM/F12 containing 1% antibiotics and 2% fetal bovine serum (FBS) (by the sample weight, the ratio of 100 mg per 1 mL) and, for extraction over either 24 h (diurnal extraction) or over 7 days (7-day extraction), placed in a CO_2_ incubator under standard conditions.

At 24 h before use with the extracts to be assayed, the cells of a test culture of 4–6 passages were removed from the prepared flasks and seeded at 5000 cells per well onto a 96-well plate (TPP Techno Plastic Products AG, Trasadingen, Switzerland). Complete growth medium (DMEM/F12 medium with penicillin/streptomycin and 10% inactivated fetal calf serum) was used as the growth medium. The cells were cultured in a CO_2_ incubator (5% CO_2_, 37 °C, high humidity) for 24 h.

After 24 h of cultivation, the condition of the cultures in the wells was assessed using light microscopy (an inverted microscope, the Leica DMIL LED, Leica, Wetzlar, Germany), with a video camera and Leica LAS V4.13 image visualization software (Leica, Wetzlar, Germany). If the culture was in good condition (typical morphology, in a representative monolayer pattern having a fairly uniform distribution of cells over the surface of the wells), it was used for analysis.

After confirmation of the good condition of the cultures, the growth medium was removed from the wells with cells and replaced either with 200 μL of fresh growth medium for the control wells or with 200 μL of the appropriate extract or one of its dilutions in growth medium for the experimental wells. The plate was placed in a CO_2_ incubator (5% CO_2_, 37 °C, high humidity) and cultured for 72 h. After this time, the plate was removed from the incubator and 20 μL of the MTT standard solution (3-(4,5-dimethylthiazol-2-yl)-2,5-diphenyltetrazolium) at a concentration of 5 mg/mL was added to each well of the plate and returned to the incubator under standard conditions for another 4 h. After incubation, the medium was completely replaced with a DMSO solution for 5 min. After staining, the optical density was determined at 540 nm on an INFINITI F50 analyzer, Tecan photometer (Tecan Austria GmbH, Grödig, Austria). Then, the relative growth intensity was calculated and the level (rank) of cytotoxicity was determined.

The relative growth rate was determined as a percentage using the following formula:

$$\mathrm{RGR}\;=\;\frac{mean\;OD\;of\;test\;compound}{mean\;OD\;of\;control}\times 100$$

where RGR is the relative growth rate, and OD is the optical density.

The cytotoxicity level (rank) was determined according to DIN/EN/ISO 10993-5:2009 [39] No cytotoxicity was evident, as the optical density/refraction in all cases was ≥70% (rank 0–1). (Various degrees of cytotoxicity would be indicated by optical density/refraction values of <70.)

### 2.5. Assessment of Cell Interaction with Material Samples

Test cultures of human ASCs were used to assess the degree of adhesion, viability, proliferation, and migration of cells within the samples of the studied materials [40]. Cells from test cultures of 3–4 passages at a concentration of 20 × 10^3^ per ml were seeded on the surfaces of the samples and cultured in a CO_2_ incubator under standard conditions. The samples were put in the wells of 24-well culture plates (Costar, Washington, DC, USA). Each matrix was examined in at least three replicates at each time point and compared with the state of culture cells seeded in three control wells directly onto the plastic. Igla medium, modified by Dulbecco (DMEM) with the addition of antibiotics (penicillin/streptomycin), glutamine, and 10% FBS, was used as the growth medium.

The cells were cultured on the samples for up to 7 days, with the medium being replaced twice during the cultivation process. At the test intervals (24 h, 3 days, and 7 days), three samples of each material were selected for fluorescence microscope examination. For each test, the cells on the samples and those in the three control wells were stained and observed using fluorescence microscopy performed on a Cytation 5 imager (BioTek, Winooski, VT, USA) with the Gen 5 Imedge+ software.

Cell nuclei were fluorescent, having been treated with Hoechst 33342 fluorochrome intravital stain (BD Biosciences™, Franclin Lakes, NJ, USA) (excitation wavelength 377 nm and emission wavelength 447 nm), while Calcein AM (BD Pharmingen™, Franclin Lakes, NJ, USA) intravital staining was used to label the cytoplasm of viable cells that had adhered to the sample materials, and to assess their morphology. The action of this dye is associated with esterase activity, which is typical only of viable cells. The staining with these fluorochromes was conducted in accordance with the manufacturers’ protocols.

Quantitative assessment of changes in the cell density on the samples during culture growth was conducted using an original technique [47]. At each test interval, the samples of matrix seeded with cells were stained with fluorochromes (see below), and the live/dead cell nuclei on the material were photographed. This technique was based on intravital staining of nuclei of living cells using Hoechst 33342 fluorochrome (Franclin Lakes, NJ, USA), and TO-PRO™3 Ready Flow™ Reagent Invitrogen™ (Walthman, MA, USA) fluorochrome, which stains the nuclei of dead cells only (excitation wavelength 586 nm, emission wavelength 647 nm). The latter was used to label and assess the proportion of dead cells in the structure of the material during cultivation. This fluorochrome has specificity for double-stranded DNA, but cannot penetrate the cytoplasm of viable cells. Wide-field fluorescence microscopy was used over 10 fields of view using the Z-stack function with subsequent counting of the live/dead cell nuclei on the linked Z-stack photomicrographs. Therefore, the total number of live/dead cells could be estimated per 1 mm^3^.

The extent of cell migration within the structure of the matrices was assessed using an original technique [48]. To implement the technique, the cell distribution at different depths in the structure of the samples was photographed in 12 fields of view with layer-by-layer shooting along the Z axis to the maximum visualization depth from the first cell (on the surface) to the last (within the thickness of the material). In other words, the depth of penetration of nuclei into the structure of the sample was recorded to represent the presence of the cells containing them.

### 2.6. Statistical Analysis

Statistical analysis was conducted using the STATISTICA 6.0 software system (TIBCO Software Inc., Palo Alto, CA, USA). Nonparametric statistics methods and the Wilcoxon paired comparison criterion were used in the study.

## 3. Results

The study was conducted using samples of the following three matrices of different origin and composition: an allogeneic matrix, a commercial osteoplastic BioOst matrix based on xenogenic bone, and a synthetic hybrid polymer material.

### 3.1. Morphology of the Bone-Substitute Materials

#### 3.1.1. Allogeneic Matrix Material

**Figure 1:**
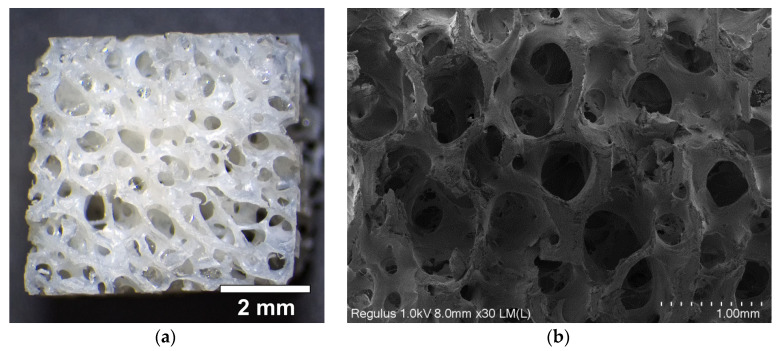
Morphology of the allogeneic matrix bone substitute: (**a**) surface photomicrograph and (**b**) SEM image of the split.

**Figure 2:**
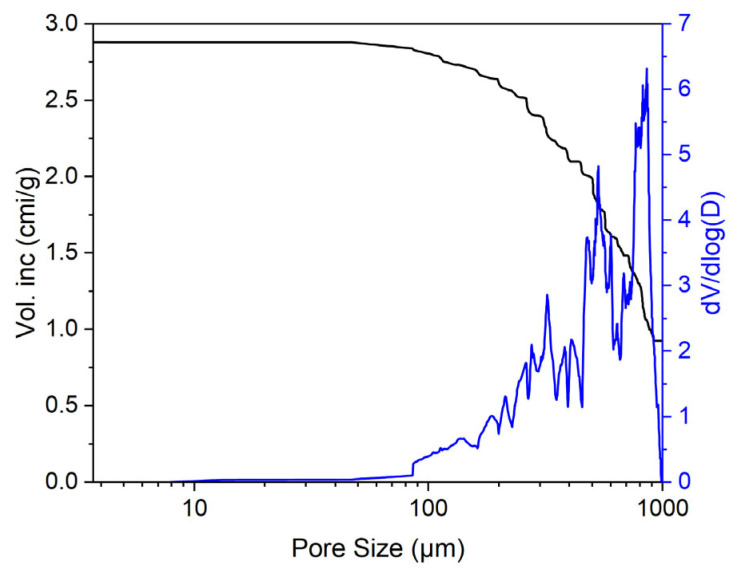
Curve of pore distribution by size within the allogeneic matrix bone substitute.

The allogeneic matrix included decellularized and deproteinized bone tissue. The samples used were square blocks with dimensions of 8 × 8 × 4 mm. The blocks were opaque and yellowish, and had visible pores (Figure 1a). The SEM image of a typical block (Figure 1b) clearly shows that the porous structure was composed of large pores ranging in size from 100 μm to 1 mm. The pores were round in cross-section and there were visible areas of intersection of several pores, indicating their interconnection. This was also confirmed by mercury porosimetry results. The porogram of the material (Figure 2) shows that 94% of the pore volume comprised pores ranging in size from 100 to 1000 μm. The size of the remaining pores was 10–100 μm. No pores smaller than 10 µm were detected.

#### 3.1.2. BioOst Osteoplast Matrix Material

**Figure 3:**
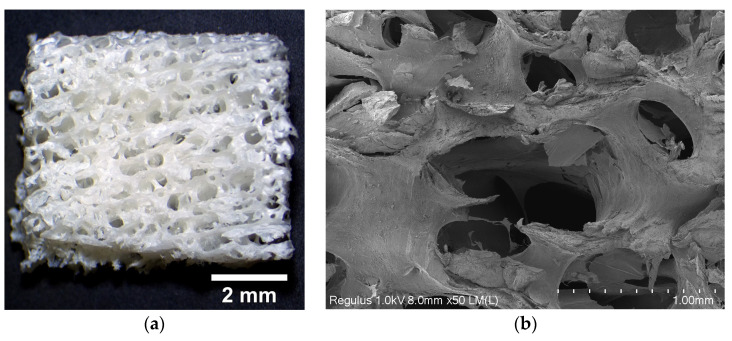
Morphology of the osteoplastic matrix BioOst bone substitute: (**a**) surface photomicrograph and (**b**) SEM image of the split.

**Figure 4:**
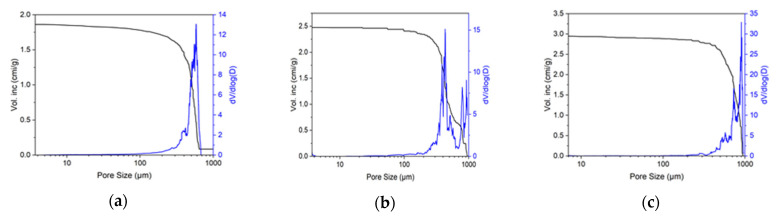
Curve of pore distribution by size within the osteoplastic matrix BioOst bone substitute: (**a**) block 1, (**b**) block 2, (**c**) block 3.

Commercial BioOst osteoplast matrix material is obtained using a proprietary process based on purified bone tissue from young cattle, subjected to strict veterinary control. The samples of the study material were opaque white blocks with visible pores. Figure 3a,b show a photomicrograph and an SEM image of the BioOst osteoplast matrix material, respectively. The material was characterized by large interconnected pores, the size of which, according to SEM, was in the order of hundreds of microns. The section had uneven pore boundaries—while some were round, others were ellipsoid or of more complex shape. Taking into account the major heterogeneity of the material’s porous structure, porograms of three blocks were obtained (Figure 4a–c).

The porosimetry demonstrated that all the blocks were characterized by a small content of pores up to 100 μm in size, while the majority of pores ranged in size from 200 to 1000 μm. In this range, the porograms of the different blocks varied significantly. For instance, the pore size of block No. 1 (Figure 4a) did not exceed 800 μm, and the majority of pores ranged from 200 to 750 μm; one peak with a maximum at 650 μm was observed on the porogram. Block No. 2 (Figure 4b) had a maximum pore size of 1000 μm. Several peaks concentrated in two areas were observed on the porogram. The first area had pores from 200 to 600 μm with the maximum values in the range 350–450 μm; the second area had pores from 600 to 1000 μm, with the maxima at 800 to 900 μm. In contrast to block No. 2, block No. 3 (Figure 4c) had all peaks concentrated in the 600–1000 µm region with maxima in the range from 700 to 900 µm. Compared to block No. 2, where they were predominant, there were a significantly smaller proportion of pores ranging from 200 to 600 μm in diameter. For three samples of the BioOst material, block 1, block 2, and block 3, the open porosity was 75, 78, and 80%, respectively. The results of comparing one block of the material to another reflected the heterogeneity of the porous structure of the BioOst osteoplastic matrix material. Summarizing the data on the porous structure of the various samples of the BioOst osteoplastic matrix material, overall, the material contained an insignificant number of pores of 50–200 µm. The majority of the pores were sized from 200 to 1000 µm, while the maximum of the pore size distribution was in the range of 350–900 µm.

#### 3.1.3. Hybrid Polymer Material

**Figure 5:**
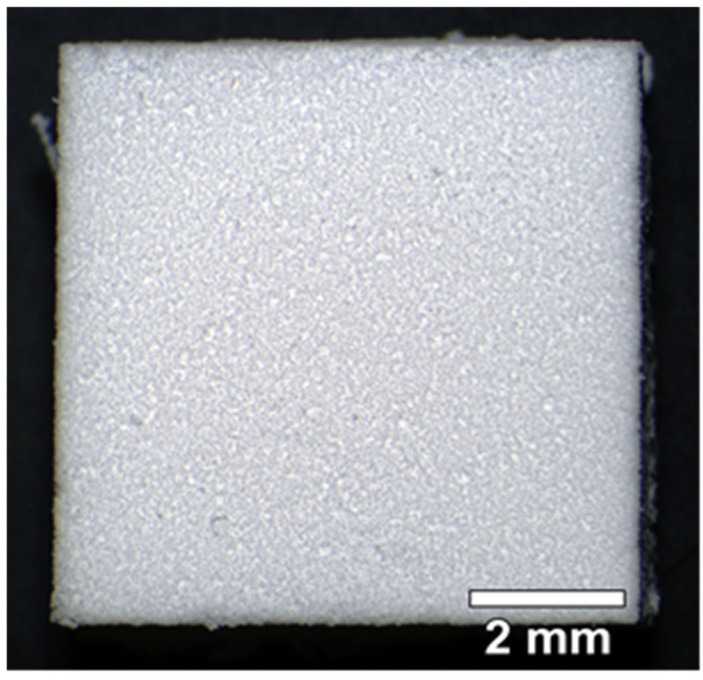
Micrograph of the surface of a sample of hybrid polymer material.

For our research, the experimental hybrid polymer material was obtained from a porous polyoligomer matrix by applying a layer of polylactide to the surface of the pores [49]. As with the other samples, the blocks had dimensions 8 × 8 × 4 mm. The photomicrograph of the material (Figure 5) showed the surface to be uneven, rough, and to have fine pores.

**Figure 6:**
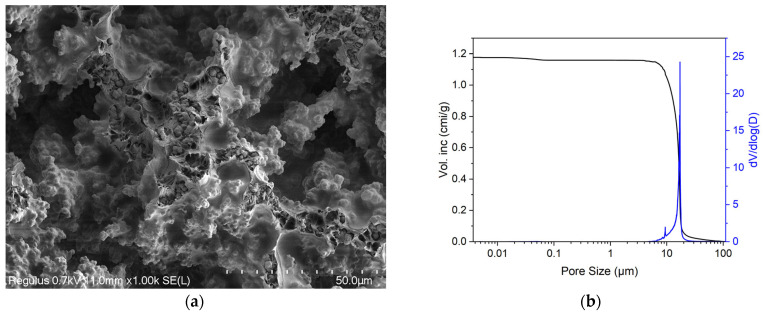
(**a**) SEM image of the cleavage surface of the hybrid polymer; (**b**) porogram of the experimental material of the hybrid polymer.

The SEM image of the polymer hybrid material section (Figure 6a) demonstrated that the material was a coral-like porous agglomeration of globular particles of 1–2 μm. The size of the agglomerates varied from 3 to 5 μm. Their surfaces were covered with a layer of polylactide. Here, the pores were the free spaces between the agglomerates. Accordingly, the pores had a complex geometry in section, where the pore size ranged from single digits to tens of microns. These data were consistent with the results of the mercury porosimetry study of the hybrid polymer matrix. The porogram (Figure 6b) showed that the pore size in the material varied from 0.7 to 100 μm. In fact, the pore size distribution was quite narrow—the biggest proportion of the pores had sizes from 10 to 20 μm with a maximum of 17 μm.

When comparing the pore characteristics of all three studied samples, while it is evident that all the materials were porous, the range of pore sizes of the materials obtained from biological tissues (allogeneic matrix and BioOst matrix) and the experimental hybrid polymer material differed significantly. While the hybrid polymer material had pores only up to 100 μm, the pore size of the biological materials ranged considerably upwards from 50 μm. Thus, the average pore size of the hybrid polymer samples was in the range of 10–20 μm, whereas those of the biological material were principally in the hundreds of microns.

The samples of the allogeneic matrix material had the highest porosity. This was evidenced by the maximum values of the specific pore volume and the total and open porosity (Table 1). The values thereof are lower for the BioOst osteoplastic matrix material. The samples of these materials had an open porosity of 79% and 75%, respectively. The pore characteristics of the hybrid polymer material were lower; the material’s open porosity was at a level of 50% (Table 1).

Physical and mechanical compression tests of the allogeneic matrix samples were performed in two load application variants: from the top and from the side (Table 1). The E value was greatest for the allogeneic matrix samples, slightly smaller for the hybrid polymer samples, and smallest for the BioOst matrix. The σ values for the samples of all the studied materials were similar. It should be noted that the compressive strength values for all the matrices were in a fairly narrow range from 4.8 to 5.8 MPa (Table 1), which corresponds to the typical strength of cancellous bone (2–45 MPa) [50].

### 3.2. Biological Characteristics of the Samples

The cytotoxicity study conducted on all samples using the MTT assay showed no cytotoxicity at either extraction time (1 and 7 days) nor at any dilution of the extracts. For all samples, the viability was ≥70%.

**Figure 7:**
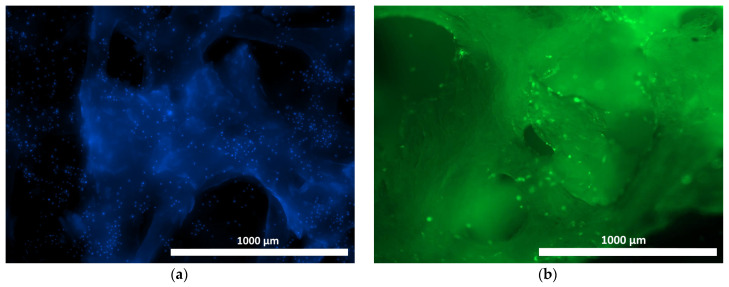
ASCs on the surface of the allogeneic matrix material (cross-linked image) after 24 h of cultivation using fluorescence microscopy: (**a**) Hoechst 33342 fluorochrome; (**b**) Calcein AM fluorochrome (40× magnification).

**Figure 8:**
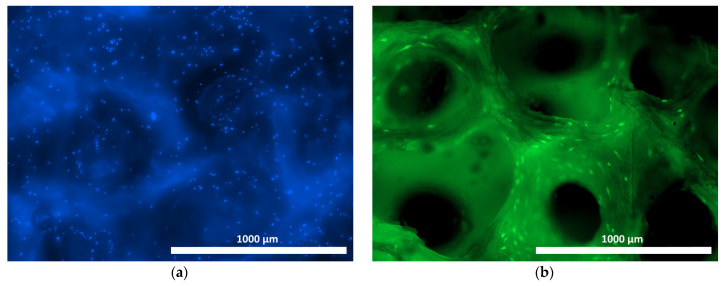
ASCs on the surface of the osteoplastic matrix BioOst material (cross-linked image) after 24 h of cultivation using fluorescence microscopy: (**a**) Hoechst 33342 fluorochrome; (**b**) Calcein AM fluorochrome (40× magnification).

**Figure 9:**
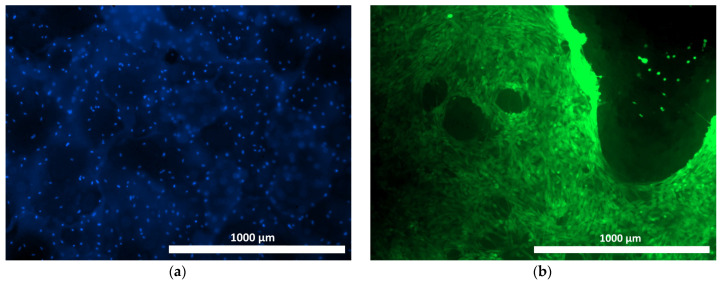
ASCs on the surface of the hybrid polymer material (cross-linked image) after 24 h of cultivation using fluorescence microscopy: (**a**) Hoechst 33342 fluorochrome; (**b**) Calcein AM fluorochrome (40× magnification).

**Figure 10:**
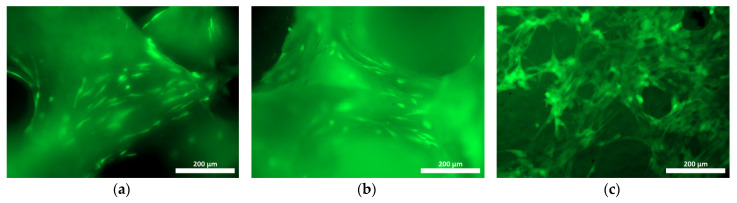
Viable ASCs on the samples of material (cross-linked image) after 48 h of cultivation using fluorescence microscopy with a Calcein AM fluorochrome (100× magnification): (**a**) on the allogeneic matrix material sample; (**b**) on the xenogenic BioOst osteoplastic matrix sample; (**c**) on a sample of the hybrid polymer.

When culturing human ASCs on samples of the materials under study, distinct cell adhesion was recorded, both on the surface and within the structure of the samples (Figure 7, Figure 8 and Figure 9). The use of Hoechst 33342 fluorochrome demonstrated a fairly uniform distribution of test culture cell nuclei on all the material samples (Figure 7a, Figure 8a and Figure 9a). The use of Calcein AM fluorochrome, which fluoresces in the cytoplasm of viable cells only, allowed us to demonstrate a high proportion of viable cells on all the test samples (Figure 7b, Figure 8b and Figure 9b). At 100× magnification, it could be observed that the cells of the test cultures were not only viable but had also spread out on the material samples while retaining the typical morphology of mesenchymal cells—a spindle shape with pronounced processes (Figure 10a–c).

**Figure 11:**
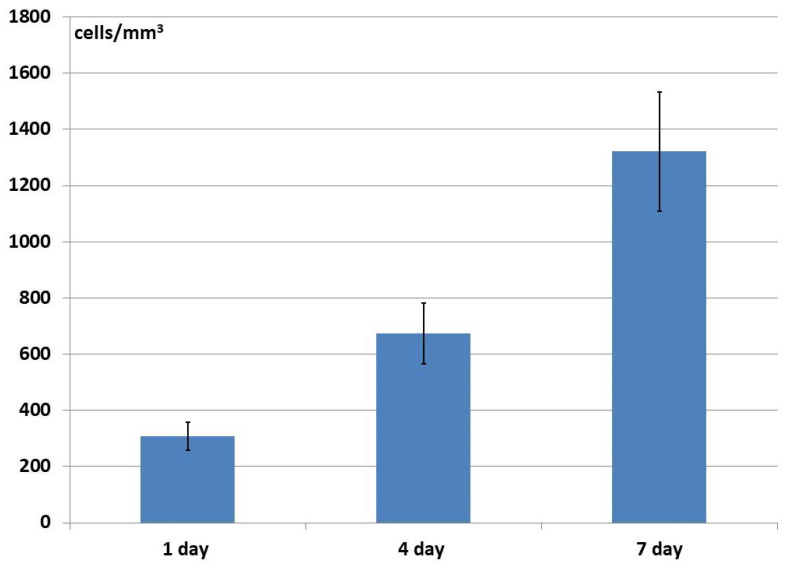
Change in the number of ASCs within the structure of the allogeneic matrix material during cultivation.

**Figure 12:**
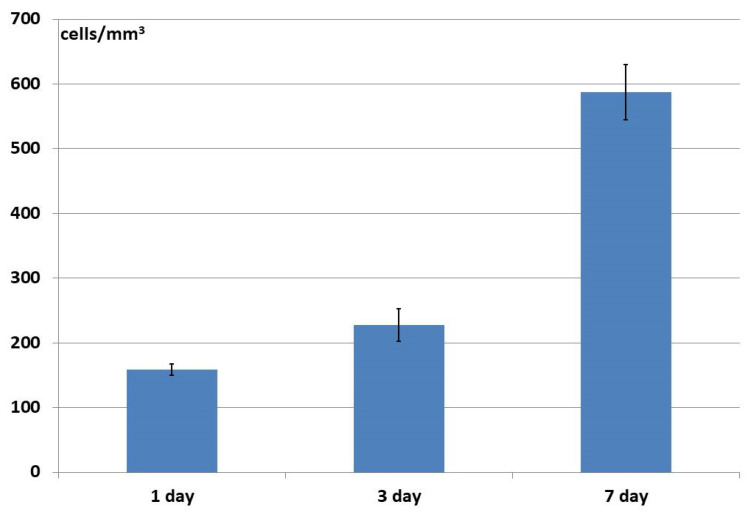
Change in the number of ASCs within the structure of the osteoplastic matrix BioOst material during cultivation.

**Figure 13:**
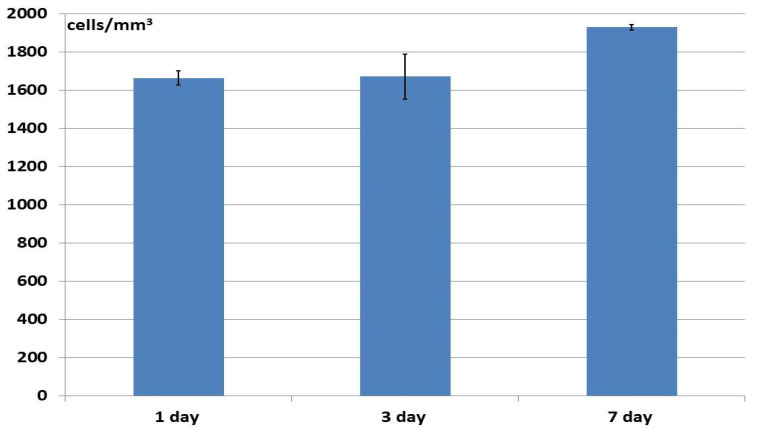
Change in the number of ASCs within the structure of the hybrid polymer material during cultivation.

Analysis of the changes in cell density on the samples during cultivation over time revealed that the number of cell nuclei increased both on the surfaces and within the structures of samples of all the studied materials (up to 7 days of observation) (Figure 11, Figure 12 and Figure 13), which proved active cell proliferation was occurring. The proportion of dead cells was insignificant and amounted to a maximum of 2–3% for each type of matrix. However, the dynamics of growth on the different materials did show variation.

For instance, the number of cells within the structure of the allogeneic matrix increased at a steady rate throughout the entire observation period (7 days), the rate of increase being statistically significant between the observation times (Figure 11). At that, the number of cell nuclei fixed within the samples by 24 h after seeding was insignificant. It can be assumed that when seeding cells onto the allogeneic matrix samples, a fairly large number of cells passed right through the large pores, therefore not becoming affixed to the samples but settling on the plastic on which they rested. A large number of ASCs on the plastic near the samples could be seen microscopically. However, the cells that did settle on the matrix surfaces or within their pores subsequently proliferated and distributed themselves within the structure of the samples, with this being subsequently observed (Figure 11).

After 24 h of cultivation, the number of cells within the structure of the xenogenic BioOst osteoplast matrix samples was not as high as in the allogeneic material, and even though numbers later increased, they did not reach such high values as in the allogeneic matrix (Figure 12). After the same initial 24 h cultivation period, a significantly higher cell content was seen in the hybrid polymer samples than in the biological materials. However, over the next three days, no further increase was recorded, but by Day 7 of observation, a statistically significant increase in their numbers had occurred and the result was comparable to the number of cells in the allogeneic material at the same time point (Figure 13).

**Figure 14:**
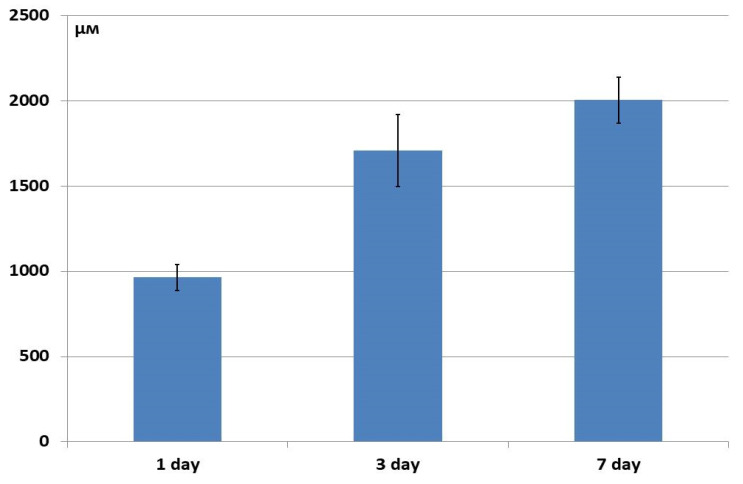
Change in the depth of penetration of ASCs into the structure of the allogeneic matrix samples.

On determining the depth of cell penetration into the sample structures, it was found that this indicator also differed, apparently being determined by the material structure and pore sizes. With the allogeneic matrix, the ASCs had become affixed at significant depths (about 900 μm) after 24 h, and by Day 3, they had penetrated statistically significantly deeper, going on to reach a depth of >2000 μm by Day 7 (Figure 14).

**Figure 15:**
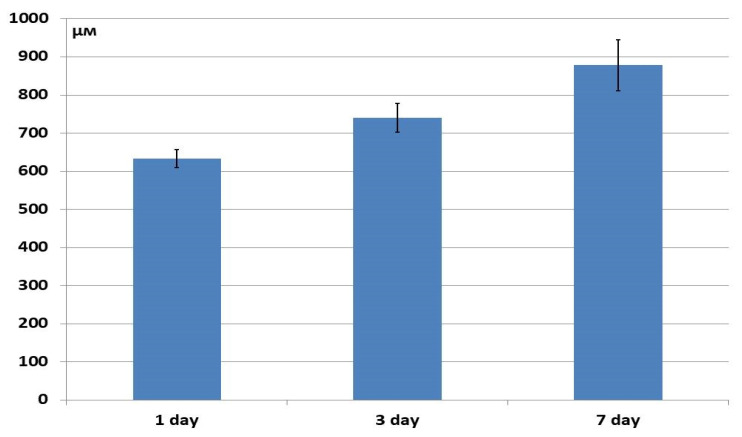
Depth of ASC penetration into the structure of xenogenic BioOst osteoplast matrix samples.

The depth of ASC penetration into the structure of the xenogenic material samples (BioOst osteoplastic matrix) after 24 h was significantly lower than for the allogeneic matrix, although they continued to move inwards through Day 3, statistically significantly increasing their penetration to 900 μm by Day 7 of cultivation (Figure 15).

**Figure 16:**
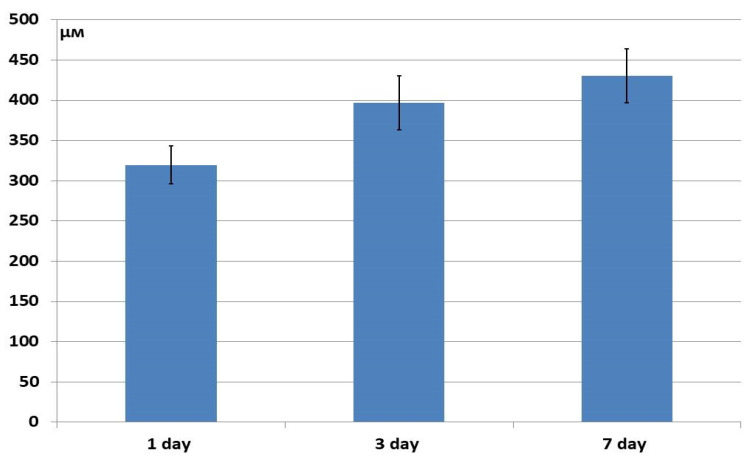
Depth of ASC penetration into the structure of hybrid polymer samples.

In the hybrid polymer samples, after 24 h of cultivation, the ASCs had penetrated the structure to 319 μm, then continued to move slightly deeper by Day 7 (Figure 16). In general, however, the depth of cell penetration into the structure of the hybrid polymer samples was quite limited. It did not exceed 450 μm, this being less, even, than the initial depths seen in the osteoplastic matrix and allogeneic matrix samples.

Overall, then, the study demonstrated that, while the matrix materials of various origin were not cytotoxic, there were specific differences in their pore characteristics and in aspects of their physical and mechanical properties. It was shown that the cells of the test cultures adhered well and spread out over the surfaces of samples of all the studied materials while preserving their typical morphology. They actively proliferated during the observation period and migrated into the structure of the samples. Both the initial and subsequent migration depths were apparently determined by the pore sizes and the structure of the materials. The in vitro data obtained indicate that the properties of the different matrices created favorable conditions for the initial stage of interaction with surface-dependent cells and for continuation of their cellular processes, both on the surface and within the structure of the materials—that is, they were all cytocompatible.

It should be remembered that the hybrid polymer essentially differed from the biological materials in its predominance of small pores with diameters of ~1–100 μm. It is widely recognized that pore characteristics are amongst the most important features contributing to successful biointegration of a bone-substitute implant in the body. The overall porosity, size, and shape of the pores of such materials and of biotissues are therefore critical for tissue engineering purposes. The pores are important in ensuring cell colonization and tissue vascularization. In this respect, the overall porosity and pore sizes in the matrices may need to be different, depending on purpose and location (as with natural bone structures in the body). For example, it is known that cortical bone has a porosity of about 5–10%, while cancellous bone’s porosity is 75–90% [51]. The importance of pores of different sizes for successful use of bone-substitute materials has been described in several studies [52,53].

Some researchers believe that to ensure cell invasion, the pore size should be in the range of at least 200–350 μm [54,55]. In other studies, though, researchers have emphasized that an interconnected microporosity in the range of 0.1–100 μm, typical of the hybrid polymer samples, is required biologically as it improves the resorption of the framework material and promotes osteogenesis [56].

Since existing materials of biological origin have disadvantages in the variability of their porous characteristics and physical and mechanical properties, in addition to the impossibility of precisely programming specified shapes or making implants from such materials using 3D printing, it can be assumed that the use of matrices based on polymeric materials should provide greater stability of properties, predictability of results, and range of applicability of their use in various clinical situations. Such reliability should be possible to ensure through careful choice of the chemical composition of these polymeric materials, offering the ability to adjust the porosity and in vivo decomposition rate. Moreover, the advantage of polymer matrices is their potential production in large quantities under controlled conditions, their long shelf life, ease of processing, and often lower cost compared to biological scaffolds [57].

## 4. Conclusions

Thus, the cytocompatibility, lack of cytotoxicity, and porous structures of all the studied materials allow us to suggest that all three, the allogeneic matrix, the xenogenic BioOst matrix, and the hybrid polymer material, are appropriate for use as matrices for biomedical research and in the formation of model constructs for bone tissue replacement.

Undoubtedly, preclinical studies of each new compound intended for biomedical research must be thorough, comprehensive, and consistent, taking into account the particular purpose to which each specific material will be put when assessing the material characteristics. Adequate selection of research methods at the earliest stages of development of each material will ensure the most effective approaches for further work and subsequent use of this product. We hope that our experience working with materials of various origins will be useful to specialists developing new products for biomedicine.

## Figures and Tables

**Table 1:** Pore, physical, and mechanical characteristics of the studied materials.

Parameters	Allogeneic Matrix	Osteoplastic Matrix BioOst	Hybrid Polymer
Pore size (average pore size), μm	50–1000 (500–800)	50–1000 (350–900)	1–100 (10–20)
Specific pore volume, cm^3^/g.	2.52	1.86	1.18
Total porosity, %	89%	78%	67%
Open porosity, %	79%	75%	48%
E, MPa	208/127	47.7	80
σ, MPa	4.8/5.1	5.2	5.8

E—modulus of elasticity; σ—breaking stress under compression.

## Data Availability

The original contributions presented in this study are included in the article. Further inquiries can be directed to the corresponding author.
